# Prognostic and diagnostic role of PSA immunohistochemistry: A tissue microarray study on 21,000 normal and cancerous tissues

**DOI:** 10.18632/oncotarget.27145

**Published:** 2019-09-10

**Authors:** Sarah Bonk, Martina Kluth, Claudia Hube-Magg, Adam Polonski, Greta Soekeland, Georgia Makropidi-Fraune, Christina Möller-Koop, Melanie Witt, Andreas M. Luebke, Andrea Hinsch, Eike Burandt, Stefan Steurer, Till S. Clauditz, Thorsten Schlomm, Daniel Perez, Markus Graefen, Hans Heinzer, Hartwig Huland, Jakob R. Izbicki, Waldemar Wilczak, Sarah Minner, Guido Sauter, Ronald Simon

**Affiliations:** ^1^ Institute of Pathology, University Medical Center Hamburg-Eppendorf, Hamburg, Germany; ^2^ General, Visceral and Thoracic Surgery Department, University Medical Center Hamburg-Eppendorf, Hamburg, Germany; ^3^ Urology Clinic, Charite – Universitätsmedizin Berlin, Berlin, Germany; ^4^ Martini Clinic, University Medical Center Hamburg-Eppendorf, Hamburg, Germany

**Keywords:** prostate specific antigen, specificity, immunohistochemistry, prognosis marker, tissue microarray

## Abstract

To assess the prognostic and diagnostic utility of PSA immunostaining, tissue microarrays containing 17,747 prostate cancers, 3,442 other tumors from 82 different (sub) types and 608 normal tissues were analyzed at two different antibody concentrations (1:100 and 1:800). In normal tissues, PSA expression was limited to prostate epithelial cells. In prostate cancers, PSA staining was seen in 99.9–100% (1:800–1:100) primary tumors, 98.7–99.7% of advanced recurrent cancers, in 84.6–91.4% castration resistant cancers, and in 7.7–18.8% of 16 small cell carcinomas. Among extraprostatic tumors, PSA stained positive in 0–3 (1:800-1:100) of 19 osteosarcomas, 1-2 of 34 ovarian cancers, 0-2 of 35 malignant mesotheliomas, 0–1 of 21 thyroid gland carcinomas and 0–1 of 26 large cell lung cancers. Reduced staining intensity and loss of apical staining were strongly linked to unfavorable tumor phenotype and poor prognosis (*p* < 0.0001 each). This was all the more the case if a combined “PSA pattern score” was built from staining intensity and pattern. The prognostic impact of the “PSA pattern score” was independent of established pre- and postoperative clinico-pathological prognostic features. In conclusion, PSA immunostaining is a strong prognostic parameter in prostate cancer and has high specificity for prostate cancer at a wide range of antibody dilutions.

## INTRODUCTION

Prostate cancer (PCa) is the most common cancer in men. More than 70% of men at the age of 75 carry one or several cancers in their prostate. Most of these tumors remain undetected and will not generate symptoms throughout the life of affected men. However, more than 170,000 prostate cancers are annually detected in the United States and 30,000 patients die from their disease [1]. This makes prostate cancer the most commonly diagnosed cancer and the second most common cause of tumor associated death in males.

Prostate specific antigen (PSA) is the most relevant protein for the management of men with suspected or diagnosed prostate cancer. The protease PSA is exclusively produced in prostate epithelial cells [2]. It is secreted to the seminal fluid and plays a role for its liquefaction [3]. Only minor quantities of PSA reach the blood stream. The serum PSA level is largely proportionate to the quantity of prostate epithelial cells in the body [4]. An increased serum PSA level is the most common cause for prostate cancer suspicion and subsequent prostate biopsy. In men with diagnosed prostate cancer, serum PSA analysis is the most commonly used parameter to monitor disease recurrence and response to therapy.

PSA analysis is also common in pathology. Due to its perceived prostate specificity, immunohistochemical PSA analysis is routinely used to determine whether tumor bulks of unknown origin can be assigned to a prostate cancer. However, cellular PSA expression can be substantially reduced in poorly differentiated prostate cancers, which can result in PSA negative immunohistochemistry and widespread metastatic prostate cancers with very low serum PSA levels [5, 6]. It is thus not surprising that studies on cohorts of 40–2,556 prostate cancers had earlier suggested associations with unfavorable tumor features or even a prognostic role of reduced PSA levels [7–10].

Although PSA immunohistochemistry is commonly used in routine histopathological diagnosis, several issues are not satisfactorily clarified. These include: 1. Is PSA expression indeed prostate cancer specific or can PSA be (ectopically) expressed in other cancers? 2. Has the immunohistochemically determined PSA level of a cancer a prognostic impact that is substantial enough to be potentially clinically useful, and 3. To what extent is the diagnostic and prognostic role of PSA immunohistochemistry dependent on the selected experimental procedure (antibody concentration)? To answer these questions, more than 20,000 prostate cancers (including hormonally treated, castration refractory, and small cell carcinomas) as well as 3,442 other malignant and benign tumors were analyzed for PSA expression utilizing two different antibody concentrations.

## RESULTS

### Prognostic role of PSA expression in prostate cancer.

**Figure 1:**
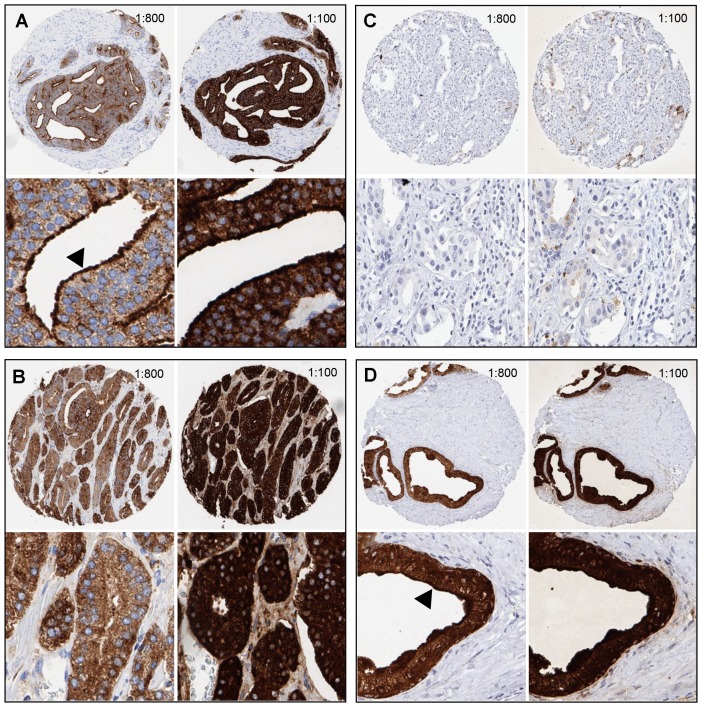
Examples of PSA immunostaining at two different antibody dilutions (1:100, 1:800) in prostate tissues. (**A**) Prostate cancer with apical staining (arrowhead). (**B**) Absence of apical staining. (**C**) PSA-negative prostate cancer. (**D**) Normal prostate glands showing apical staining (arrowhead).

**Figure 2:**
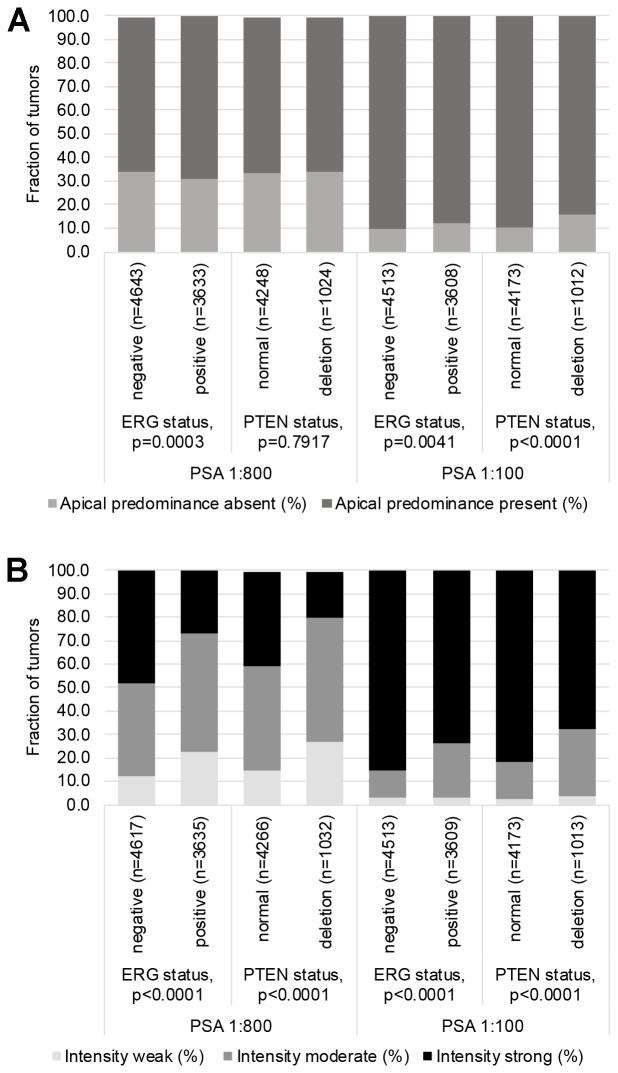
Associations between PSA immunostaining results (using the anti PSA antibody at 1:100 and 1:800 dilution), TMPRSS2: ERG fusion status and PTEN deletion status. (**A**) PSA immunostaining scored for presence or absence of apical predominance. (**B**) PSA immunostaining scored for the staining intensity.

**Figure 3:**
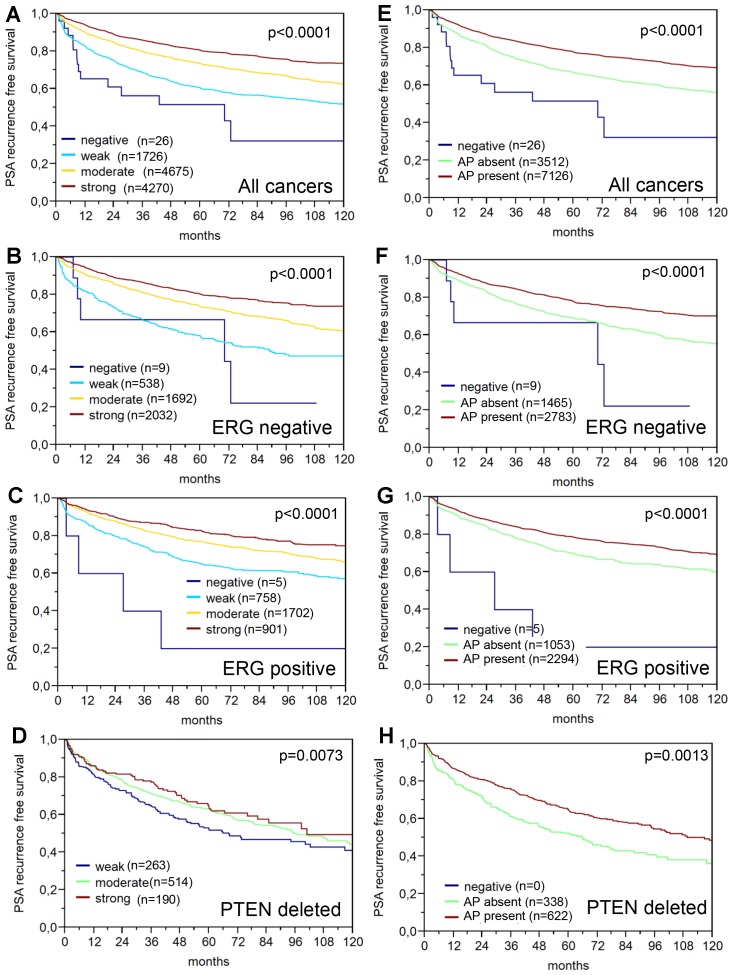
Prognostic relevance of PSA immunostaining (dilution 1:800) in prostate cancer. (**A**–**D**) Impact of the PSA staining intensity in (**A**) all cancers, (**B**) TMPRSS2: ERG, (**C**) TMPRSS2: ERG positive and (**D**) PTEN deleted cancers. (**E**–**H**) Impact of the presence or absence of apical predominance (AP) of the PSA staining in (**E**) all cancers, (**F**) TMPRSS2: ERG negative, (**G**) TMPRSS2: ERG positive and (**H**) PTEN deleted cancers.

**Figure 4:**
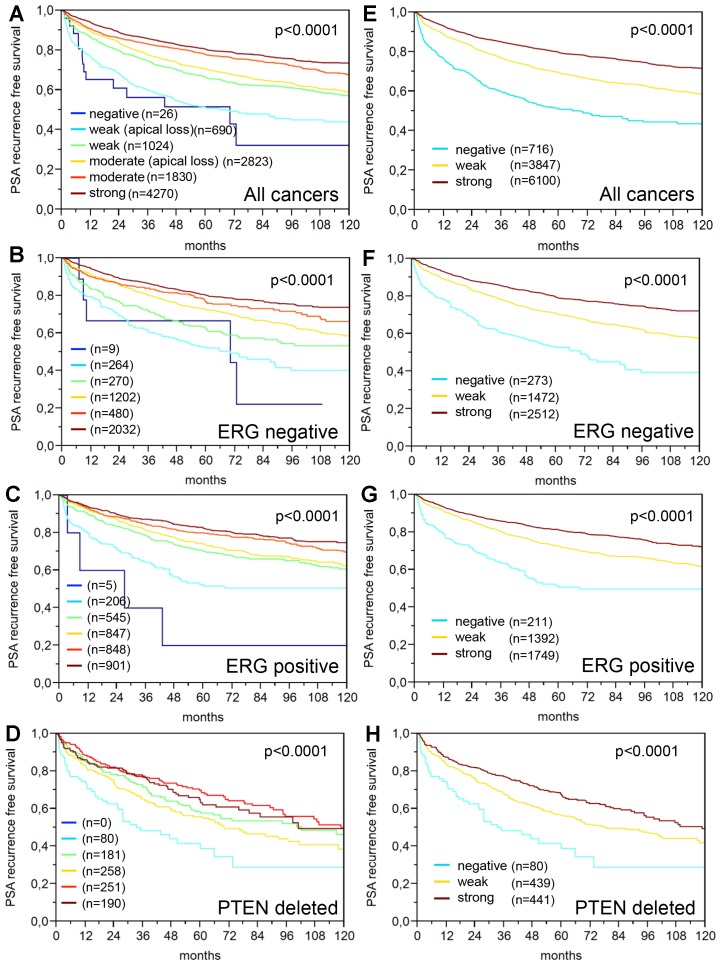
Prognostic relevance of the PSA staining in prostate cancer. (**A**–**D**) Combination of the PSA staining intensity and apical predominance in (**A**) all cancers, (**B**) TMPRSS2: ERG negative cancers, (**C**) TMPRSS2: ERG positive cancers and (**D**) PTEN deleted cancers. (**E**–**H**) Prognostic impact of the “PSA pattern score” in (**E**) all cancers, (**F**) TMPRSS2: ERG negative, (**G**) TMPRSS2: ERG positive and (**H**) PTEN deleted cancers.

64% and 62% of the 17,747 tumor samples were interpretable in our TMA analysis utilizing different (1:800 and 1:100) antibody concentrations. Reason for non-informative cases included lack of tissue samples or absence of unequivocal cancer tissue in the TMA spot. In normal prostate glands, PSA immunostaining typically showed a conspicuous predominance at the apical portion of the cells. Apical predominance was also retained in a fraction of cancers. Examples of PSA immunostainings in prostate tissues are given in Figure 1. Reduced PSA levels were associated with TMPRSS2: ERG fusions and PTEN deletions. Both reduced staining intensity and a loss of apical predominance (apical loss) of PSA staining were strikingly linked to unfavorable tumor phenotype and prognosis. This also hold true for subsets of ERG positive, ERG negative and PTEN deleted cancers. The respective data are shown for the 1: 800 dilution in Supplementary Table 1 and Figures 2 and 3. The combined analysis of PSA staining pattern and intensity demonstrated that the outcome of cancers with apical staining loss was comparable to cancers having a “one level lower” intensity score (Figure 4). Accordingly, tumors with moderate staining intensity and apical staining loss were considered “weak” and tumors with weak staining intensity and apical staining loss were considered “negative” in a separate analysis. Tumors with moderate to strong staining were combined into one group “strong”. The prognostic impact of this “*PSA pattern score*” was statistically independent of established prognostic parameters (Table 1). If the PSA antibody was diluted 1:100, the fraction of completely “PSA negative” cases decreased from 0.2% (antibody dilution 1: 800) to 0.07% and the fraction of tumors with “strong PSA positivity” increased from 40% (at 1: 800) to 81%. Irrespective of the changes in the number of cancers classified as PSA “negative”, “weak”, “moderate” and “strong”, striking and statistically independent statistical associations with tumor phenotype and patient outcome were similarly visible for pattern and intensity of PSA staining at 1: 100 (Supplementary Table 2 and Supplementary Figure 1).

**Table 1:** Multivariat analysis including established prognostic parameters and the “PSA pattern score” (PSA score)

Tumor subset	Scenario	*p* -value								
		n analyzable	preoperative PSA-Level	pT Stage	cT Stage	Gleason grade prostatectomy	Gleason grade biopsy	pN Stage	R Stage	PSA score
all cancers	1	6,923	<0.0001	<0.0001	—	<0.0001	—	<0.0001	<0.0001	<0.0001
	2	10,552	<0.0001	<0.0001	—	<0.0001	—	—	<0.0001	<0.0001
	3	10,392	<0.0001	—	<0.0001	<0.0001	—	—	—	<0.0001
	4	8,878	<0.0001	—	<0.0001	—	<0.0001	—	—	<0.0001
ERG-negative cancers	1	2,723	0.0002	<0.0001	—	<0.0001	—	0.0008	0.0848	<0.0001
	2	4,245	<0.0001	<0.0001	—	<0.0001	—	—	0.0033	<0.0001
	3	4,206	<0.0001	—	<0.0001	<0.0001	—	—	—	<0.0001
	4	4,138	<0.0001	—	<0.0001	—	<0.0001	—	—	<0.0001
ERG-positive cancers	1	2,134	0.0225	<0.0001	—	<0.0001	—	0.2417	0.0002	0.0226
	2	3,339	0.0002	<0.0001	—	<0.0001	—	—	<0.0001	0.0295
	3	3,282	<0.0001	—	<0.0001	<0.0001	—	—	—	0.0042
	4	3,229	<0.0001	—	<0.0001	—	<0.0001	—	—	<0.0001

For definition of the scenarios, see Statistics section.

### Diagnostic role of PSA immunostaining

**Figure 5:**
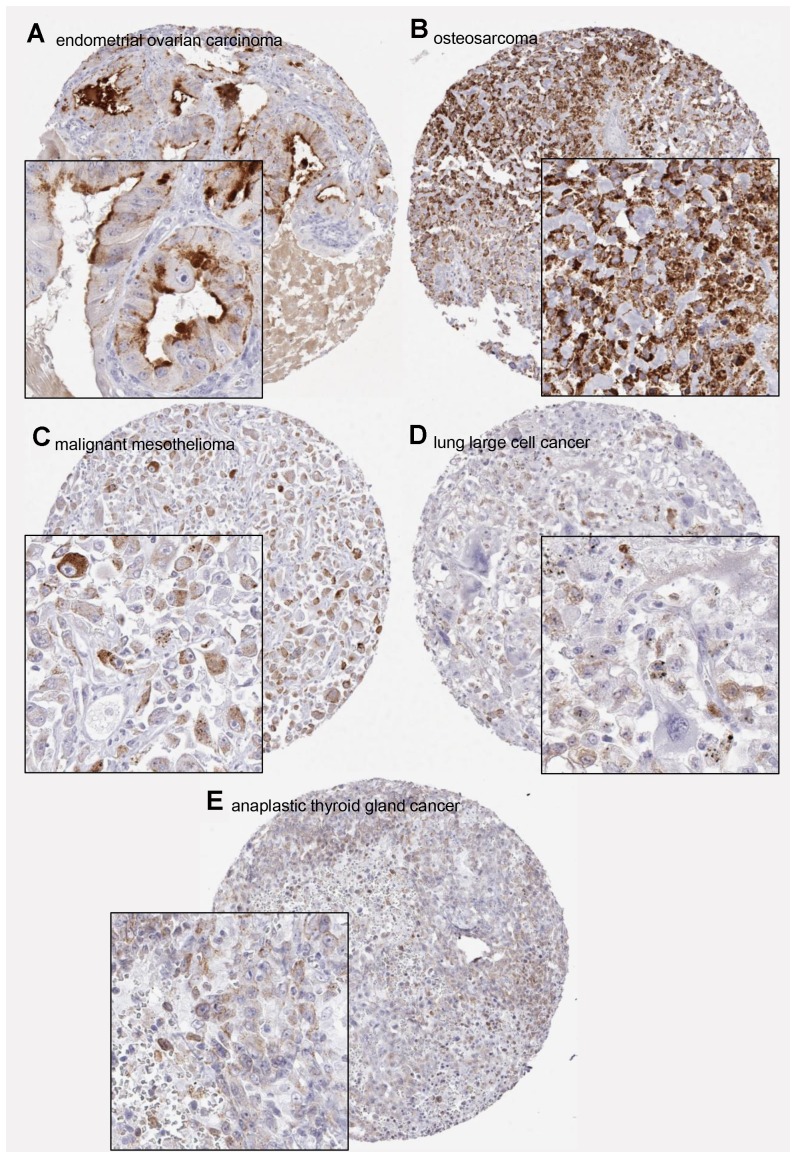
Examples of positive PSA immunostainings in non-prostatic tumors. (**A**) Anti-PSA antibody dilution 1:800, (**B**–**E**) antibody dilution 1:100.

To evaluate the sensitivity and specificity of PSA immunostaining for diagnosing prostate cancer, 12,824 prostate cancers and 2,845 tumors from other origins were evaluated at two antibody concentrations. The data from various categories of prostate cancer, and of all tumor types showing occasional PSA immunostaining are shown in Table 2. At 1:800, 99.9% of Gleason ≤3+4 show detectable PSA immunostaining. The fraction of “PSA negative” cancers increased with cancer dedifferentiation but even in case of castration refractory cancers, the rate of positivity was still >80%. However, only 1 of 13 small cell neuroendocrine carcinomas of the prostate showed PSA expression. In all these prostate cancers, the use of an eightfold higher antibody concentration increased the positivity rate. This increase was only marginally in case of Gleason ≤3+4 cancers but more significant in dedifferentiated cancers. PSA immunostaining was not completely specific for tumors of the prostate. However, only one extraprostatic cancer, i. e., an endometroid cancer of the ovary, showed detectable PSA staining at 1:800 (Figure 5A). At 1:100, PSA positivity was seen in additional 8 (total: 9 of 2,845, 0.3%) interpretable extraprostatic cancers, including another ovarian cancer, 3 osteosarcomas, 2 malignant mesotheliomas, and one case each of thyroid gland cancer and large cell lung cancer (Figure 5B–5E). A list of PSA negative cancers is given in Table 3. Examples of PSA immunostainings are shown in Supplementary Figure 2. All eight normal prostatic tissues were PSA positive while PSA staining was absent in all other analyzed normal tissues including mesenchymal tissues (aorta/intima, aorta/media, heart (left ventricle), skeletal muscle, sceletal muscle/tongue, myometrium, appendix (muscular wall), esophagus (muscular wall), stomach (muscular wall), ileum (muscular wall), colon descendens (muscular wall), kidney pelvis (muscular wall), urinary bladder (muscular wall), penis (glans/corpus spongiosum), ovary (stroma), fat tissue (white)), surfaces (skin (surface), skin (hairs, sebaceous glands), lip (epithelium), oral cavity, tonsil (surface epithelium), anal canal (skin), anal canal (transition epithelium), exocervix, esophagus, kidney pelvis, urinary bladder, amnion/chorion, stomach (antrum), stomach (fundus and corpus), small intestine, duodenum, small intestine, ileum, appendix, colon descendens, rectum, gallbladder, bronchus, paranasal sinus) and solid organs (lymph node, spleen, thymus, tonsil, liver, pancreas, parotid gland, submandibular gland, sublingual gland, lip (small salivary gland), duodenum (Brunner gland), kidney cortex, kidney medulla, prostate, seminal vesicle, epididymis, testis, lung (parenchyma), lung (bronchial glands), breast, endocervix, endometrium (proliferation), endometrium (secretion), fallopian tube, endometrium (early decidua), ovary (stroma), ovary (corpus luteum), ovary (follicular cyst), placenta (first trimester), placenta (mature), adrenal gland, parathyroid gland, thyroid, cerebellum, cerebrum, pituitary gland (posterior lobe), pituitary gland (anterior lobe)).

**Table 2:** Sensitivity and specificity of DIA-PSA at 1:100 and 1:800 antibody dilution

	Analyzable (*n*)		PSA positive (%)	
	PSA antibody concentration		PSA antibody concentration	
	low (1:800)	high (1:100)	low (1:800)	high (1:100)
**Prostate cancers**				
Gleason ≤3+4	9934	9672	99.89	99.96
Gleason 4+3	2226	2190	99.64	99.95
Primary ca. ≥8	233	216	98.71	99.07
Recurrent ca. ≥8	392	383	98.72	99.74
CR ca., Gleason ≥8	26	35	84.62	91.43
Small cell cancers	13	16	7.69	18.75
*Total*	*12824*	*12512*	*99.66*	*99.81*
**Non-prostate cancers**				
Osteosarcoma	19	19	0	15.79
Ovary, endometroid ca.	30	34	3.33	5.88
Malignant Mesothelioma	37	39	0	5.71
Thyroid gland, anaplastic ca.	24	23	0	4.76
Lung, large cell ca.	38	39	0	3.85
Other cancers types	2697	2671	0	0
*Total*	*2845*	*2825*	*0.04*	*0.32*

**Table 3:** Tumor types staining negative with DIA-PSA

Organ system	Tumor type	n (on TMA)	n (analyzable)
Skin	Pilomatrixoma	35	23
	Basalioma	48	44
	Benign naevus	29	22
	Skin squamous cell cancer	50	39
	Malignant melanoma	48	44
	Merkel cell cancer	46	46
Respiratory tract	Larynx squamous cell cancer	50	32
	Oral cavity squamous cell cancer	50	35
	Lung squamous cell cancer	50	36
	Lung adenocarcinoma	50	33
	Lung bronchioalveolary carcinoma	6	5
	Lung small cell carcinoma	13	16
	Salivary gland pleomorphic adenoma	50	31
	Salivary gland Warthin tumor	49	43
	Salivary gland basal cell adenoma	15	12
Femal genital tract	Vagina squamous cell cancer	48	45
	Vulva squamous cell cancer	50	41
	Cervix squamous cell cancer	50	49
	Cervix adenocarcinoma	50	44
	Endometrial carcinoma serous	50	46
	Uterine stroma sarcoma	12	8
	Carcinosarcoma	48	39
	Ovarian cancer endometroid	37	34
	Ovarian cancer serous	50	45
	Ovarian cancer mucinous	26	21
	Brenner tumor	9	7
	Breast cancer of no special type	46	33
	Breast cancer lobulary	43	34
	Breast cancer medullary	15	13
	Breast cancer tubulary	18	13
	Breast cancer muzinous	22	15
	Breast cancer phylloid	50	33
Gastrointestinal tract	Colon adenoma, low grade	50	46
	Colon adenoma, high grade	50	50
	Colon adenocarcinoma	50	42
	Small intestine adenocarcinoma	10	6
	Gastric cancer, diffuse	50	33
	Gastric cancer, intestinal	50	39
	Esophageal adenocarcinoma	50	29
	Esophageal squamous cell cancer	49	37
	Anal canal squamous cell cancer	50	46
	Cholangiocellulary carcinoma	50	45
	Hepatocellulary carcinoma	50	50
	Pankreatic ductal adenocarcinoma	50	32
	Pankreatoc papilla adenocarcinoma	30	19
	Pankreatic neuroendocrine tumor	49	46
	Gastrointestinal stroma tumor (GIST)	50	46
Male urogenital tract	Urinary bladder cancer pTa	50	31
	Urinary bladder cancer pT2-4	50	39
	Urinary bladder cancer small cell	18	18
	Renal cell carcinoma clear cell	50	40
	Renal cell carcinoma papillary	50	35
	Renal cell carcinoma chromophobic	50	42
	Oncocytoma	50	38
	Prostata cancer	49	47
	Prostata cancer small cell	17	16
	Seminoma	50	42
	Embryonal carcinoma (testis)	50	35
	Yolk sack tumor	50	33
	Teratoma	50	22
Endocrine system	Thyroid adenoma	50	47
	Thyroid cancer papillary	50	47
	Thyroid cancer folliculary	49	45
	Thyroid cancer medullary	50	39
	Adrenal gland adenoma	50	40
	Adrenal gland carcinoma	26	26
	Phaeochromocytoma	50	49
	Neuroendocrine tumor (NET)	50	39
Lymphatic system	Hodgkin’s-lymphoma	45	32
	Non Hodgkin’s-lymphoma	48	39
	Thymoma	29	21
Soft tissue	Granular cell tumor	30	24
	Giant cell tumor of the tendon sheat	45	43
	Leiomyoma	50	41
	Leiomyosarcoma	49	39
	Liposarcoma	49	37
	Angiosarcoma	32	25
Bone	Chondrosarcoma	25	9

## DISCUSSION

The data from this study demonstrate that PSA measurement, apart from its known high sensitivity and specificity for prostatic epithelial tissue, provides striking prognostic information in prostate cancer patients.

The immunohistochemical analysis of protein expression is subject to inherent limitations. The staining intensity and its signal to noise ratio is markedly dependent from the type of reagents and the applied experimental protocols. Accordingly, literature data on the immunohistochemically detected expression are highly variable for most proteins that have been analyzed by different research groups [17, 18]. The relatively small range, where protein expression can be quantitated in brightfield immunohistochemistry contributes to this problem. The selected experimental procedure defines an expression range below of which all staining will be “negative” and above of which all staining results will be “strongly positive”. If an immunostaining results in “dark brown” tissue elements, a tenfold higher concentration of the protein of interest will no longer lead to a discernibly stronger staining. To minimize the risk that our experimental procedure will result in particularly good or bad data just because we were lucky (or not) to select a suitable protocol we performed the prostate cancer prognosis study by using two different antibody concentrations differing by a factor of 8.

Overall these data show that the PSA expression level in prostate cancer cells is one of the strongest prognostic features in this tumor entity. This is not only demonstrated by the independent prognostic value of PSA staining in several models but also by its strong prognostic impact in PTEN deleted cancers. PTEN deletion is another highly prognostic feature, which has recently been recommended for measurement in routine praxis by several authors [19–22]. Most prognostic biomarkers lose their prognostic impact in the subgroup of PTEN deleted cancers which already are characterized by a poor prognosis [23, 24]. The reason for higher tumor aggressiveness in cancers with reduced PSA expression is unclear. Some authors have suggested a tumor protective role of PSA. For example, Heidtmann et al. showed that PSA exerts antiangiogenic properties by converting Lys-plasminogen to biologically active angiostatin-like fragments [25]. Gkika et al. found that PSA reduces motility of PC-3 prostate cancer cells through stimulation of a particular ion channel at the plasma membrane [26]. Bindukumar et al. reported that PSA treatment modulated the expression of growth factors and suppressed the growth of prostate tumor xenografts in mice [27]. However, PSA production may be one of the most important functions of normal prostate glandular cells. One can thus speculate, that a measurable deficiency in this function might represent a subtle sign of cellular dedifferentiation. Normal prostatic glands exhibit a particular strong PSA staining at the apical cell border. That a loss of this physiological apical predominance of PSA staining is directly linked to poor prognosis, irrespective of the perceived overall staining intensity, is consistent with altered PSA representing “dedifferentiation”.

The successful analysis of more than 12,000 prostate cancers revealed that even in case of undifferentiated (Gleason ≥8) or castration resistant disease, more than 99% of prostate cancers expressed PSA at a level that was detectable at the higher antibody concentration. The 0.04% PSA negative Gleason ≤3+4=7 cancers are most likely due to pre-analytical tissue damage for example caused by insufficient or prolonged formalin fixation. That small cell neuroendocrine cancers were mostly PSA negative was expected based on earlier literature [28–31]. It is of note, however, that 3 of 15 small cell carcinomas significantly expressed PSA. This demonstrates that PSA immunohistochemistry can help to identify the prostatic origin in a fraction of small cell carcinomas. The analysis of more than 2,800 non prostatic tumors showed that a positive PSA immunostaining is not completely prostate-specific. It is well known, however, that cancers can ectopically express all kinds of proteins [32]. Ectopic PSA production is thus not completely surprising. Several earlier studies have reported PSA immunostaining in considerable fractions of extraprostatic normal and neoplastic tissues. PSA expression was for example found in 9%–60% breast cancers [33–38], in 6 of 11 pleomorphic adenomas of the salivary gland and in one case of salivary duct carcinoma [39, 40], in all 56 cases of normal salivary gland [41], in 100% of 62 samples obtained from normal pancreas and normal salivary glands, pleomorphic adenoma, adenocarcinoma and Warthin’s tumor [42], in individual cases of paraurethral adenocarcinoma [43–47] and urinary bladder cancer [48] as well as in 22 of 40 (55%) of malignant melanomas [49]. Our comprehensive investigation of non-prostatic tumors for PSA expression does not provide evidence for a significant specificity problem of PSA immunohistochemistry. PSA immunostaining is rare and typically weak in extra-prostatic tumors. The only extra-prostatic cancer with PSA positivity at 1:800 was a gynecological tumor. A case report on a PSA-positive endometroid ovarian cancer can also be found in the literature [50]. That a dilution of 1:800 can increase the specificity of this diagnostic test without losing significant sensitivity is valuable also with respect to economic considerations, with is a major concern in many laboratory institutions nowadays.

In summary, the comparison of two immunohistochemical protocols identifies the high antibody concentration as a suitable diagnostic approach resulting in a specificity of 99.9%, an overall sensitivity of 99.7% and a sensitivity in more demanding histologies (Gleason ≥8) of 98.7%. The data also identify PSA expression as a striking prognostic parameter. The equally strong prognostic impact of PSA measurement at two different antibody concentrations suggest that the prognostically relevant expression range of PSA is very broad. PSA expression quantification over a broader range - for example by using fluorescence - might result in even better prognostic information.

## MATERIALS AND METHODS

### Prostate cancer prognosis study

The prostate cancer prognosis TMA contained one sample each from 17,747 patients undergoing surgery between 1992 and 2015 at the Department of Urology and the Martini Clinics at the University Medical Center Hamburg-Eppendorf. All prostate specimens were analyzed according to a standard procedure, including a complete embedding of the entire prostate for histological analysis [11]. Follow-up data were available for a total of 14,667 patients with a median follow-up of 48 months (range: 1 to 276 months). Histo-pathological and clinical data are summarized in Table 4. The molecular database attached to this TMA contained results on ERG expression [12], ERG break apart FISH analysis [13] and deletion status of 10q23 (*PTEN*). ERG protein expression from 5,515 and ERG rearrangement analysis by fluorescence *in situ* hybridization (FISH) from 8,134 tumors [13, 14] and 10q23 (PTEN) deletion status from 5,158 tumors [15].

**Table 4:** Composition of the prostate prognosis tissue microarray

	No. of patients (%)	
	Study cohort on TMA	Biochemical relapse among categories
	(*n* = 17,747)	
Follow-up (mo)		
*n*	14667 (82.6%)	3612 (24.6%)
Mean	56.3	—
Median	48	—
Age (y)		
≤50	433 (2.4%)	66 (15.2%)
51-59	4341 (24.5%)	839 (19.3%)
60-69	9977 (56.4%)	2073 (20.8%)
≥70	2936 (16.6%)	634 (21.6%)
**Pretreatment PSA (ng/ml)**	
<4	2225 (12.6%)	313 (14.1%)
4–10	10520 (59.6%)	1696 (16.1%)
10–20	3662 (20.8%)	1043 (28.5%)
>20	1231 (7%)	545 (44.3%)
**pT stage (AJCC 2002)**	
pT2	11518 (65.2%)	1212 (10.5%)
pT3a	3842 (21.7%)	1121 (29.2%)
pT3b	2233 (12.6%)	1213 (54.3%)
pT4	85 (0.5%)	63 (74.1%)
**Gleason grade**		
≤3+3	3570 (18.1%)	264 (7.4%)
3+4	9336 (47.4%)	1436 (15.4%)
3+4 Tert.5	1697 (8.6%)	165 (9.7%)
4+3	2903 (14.7%)	683 (23.5%)
4+3 Tert.5	1187 (6%)	487 (41%)
≥4+4	999 (5.1%)	531 (53.2%)
**pN stage**		
pN0	10636 (89.4%)	2243 (21.1%)
pN+	1255 (10.6%)	700 (55.8%)
**Surgical margin**		
Negative	14297 (80.8%)	2307 (16.1%)
Positive	3388 (19.2%)	1304 (38.5%)

NOTE: Numbers do not always add up to 17,747 in the different categories because of cases with missing data.

Abbreviation: AJCC, American Joint Committee on Cancer.

### Normal tissue, advanced prostate cancer and multitumor TMA

The normal tissue TMA was composed of 8 samples each of 76 different normal tissue types (608 samples on one slide). Each sample was derived from a different donor. Our multi tumor TMA contained 6–50 (total: 3,442) samples each from 82 different human tumor types and subtypes [16] distributed among 8 different TMA blocks. The exact composition of the normal and multi tumor TMAs is given in the results section. To enrich for prostate cancers that are most likely to have low PSA expression, an additional “advanced prostate cancer” TMA contained tissues from 316 patients who underwent transurethral resection for recurrent and advanced prostate cancer. The cohort included 55 patients that were known to be castration resistant and 257 patients for which the cancers sensitivity to hormone withdrawal was unknown. For all TMA sets, tissue cylinders with a diameter of 0.6 mm were punched from representative tumor or normal areas of each tissue block and brought into a recipient paraffin block. All tumor samples were obtained from the archives of the Institute of Pathology of the University Medical Center Hamburg Eppendorf. The use of archived diagnostic left-over tissues for manufacturing of TMAs and their analysis for research purposes has been approved by local laws (HmbKHG, §12,1) and by the local ethics committee (Ethics commission Hamburg, WF-049/09). All work has been carried out in compliance with the Helsinki Declaration.

### Immunohistochemistry (IHC)

Freshly cut TMA sections were immunostained on one day and in one experiment. The mouse monoclonal PSA antibody (Dianova DIA-PSA, clone HAM18) was applied at 1:100 and 1:800. Slides were deparaffinized and exposed to heat-induced antigen retrieval for 15 minutes at 98°C in pH9.0 target retrieval solution (Agilent, Santa Clara, CA, USA) in a PT Link pre-treatment module (Agilent) and stained in an Autostainer Link 48 device (Agilent). Protocol steps include 5 min peroxidase blocking (Agilent REAL), 20 min of primary antibody incubation at room temperature and visualization of the bound antibody using the EnVision Flex Kit (Agilent) according to the manufacturer’s directions. Staining was typically homogenous in the analyzed tissue samples and staining intensity of all cases was semiquantitatively assessed in four categories: negative, weak, moderate, and strong.

### Statistics

Statistical calculations were performed with JMP 11.0.0 software (SAS Institute Inc., NC, USA). Contingency tables and the chi^2^-test were performed to search for associations between molecular parameters and tumor phenotype. Survival curves were calculated according to Kaplan-Meier. The Log-Rank test was applied to detect significant survival differences between groups. Cox proportional hazards regression analysis was performed to test the statistical independence and significance between pathological, molecular and clinical variables by analyzing 4 different scenarios (Table 1). Scenario 1 evaluated all postoperatively available parameters including pathological tumor stage, pathological lymph node status (pN), surgical margin status, preoperative serum PSA value and pathological Gleason grade obtained after the morphological evaluation of the entire resected prostate. In scenario 2, all postoperatively were used but nodal status was excluded as this parameter was often lacking, preferentially in low grade cancers. The scenarios 3 and 4 modeled the preoperative situation as much as possible and included preoperative PSA and clinical tumor stage (cT stage). The scenarios 3 and 4 differed in the Gleason grade, which was either obtained on the prostatectomy specimen (scenario 3) or reflected the preoperative Gleason grade defined on the original biopsy by hundreds of different pathologists.

## SUPPLEMENTARY MATERIALS






