# Supplementary material for: Prognostic and diagnostic role of PSA immunohistochemistry: A tissue microarray study on 21,000 normal and cancerous tissues
Source: Oncotarget. 2019 Sep 10;10(52):5439–53. doi: 10.18632/oncotarget.27145 (PMC6739211; doi:10.18632/oncotarget.27145)
Supplement: Supplementary file 1 [file oncotarget-10-5439-s001.pdf]

Prognostic and diagnostic role of PSA immunohistochemistry: A tissue microarray study on 21,000 normal and cancerous tissues

SUPPLEMENTARY MATERIALS

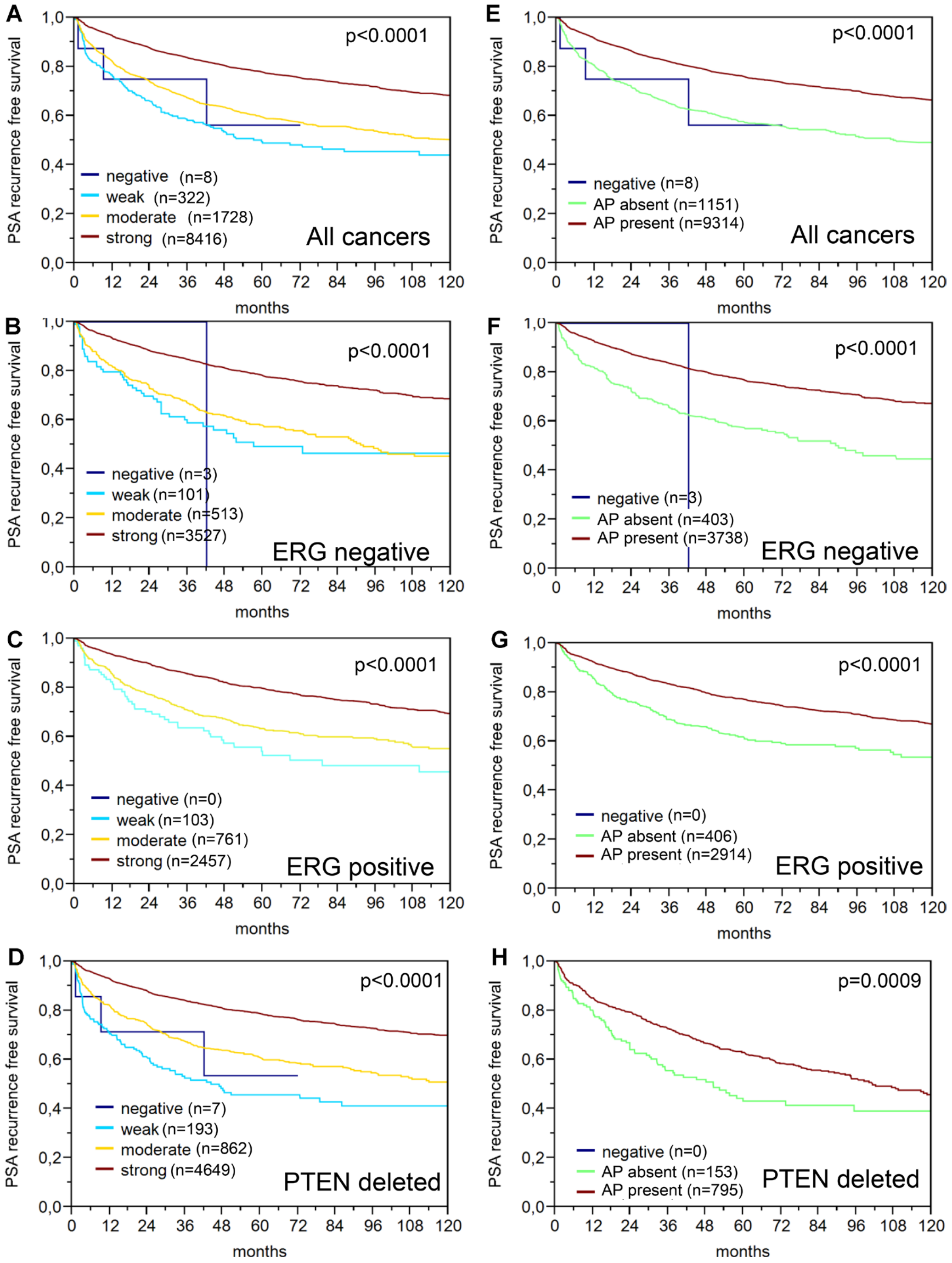

Supplementary Figure 1: Prognostic relevance of PSA immunostaining (dilution 1:100) in prostate cancer. (**A–D**) Impact of the PSA staining intensity in (**A**) all cancers, (**B**) TMPRSS2: ERG, (**C**) TMPRSS2: ERG positive and (**D**) PTEN deleted cancers. (**E–H**) Impact of the presence or absence of apical predominance (AP) of the PSA staining in (**E**) all cancers, (**F**) TMPRSS2: ERG negative, (**G**) TMPRSS2: ERG positive and (**H**) PTEN deleted cancers.

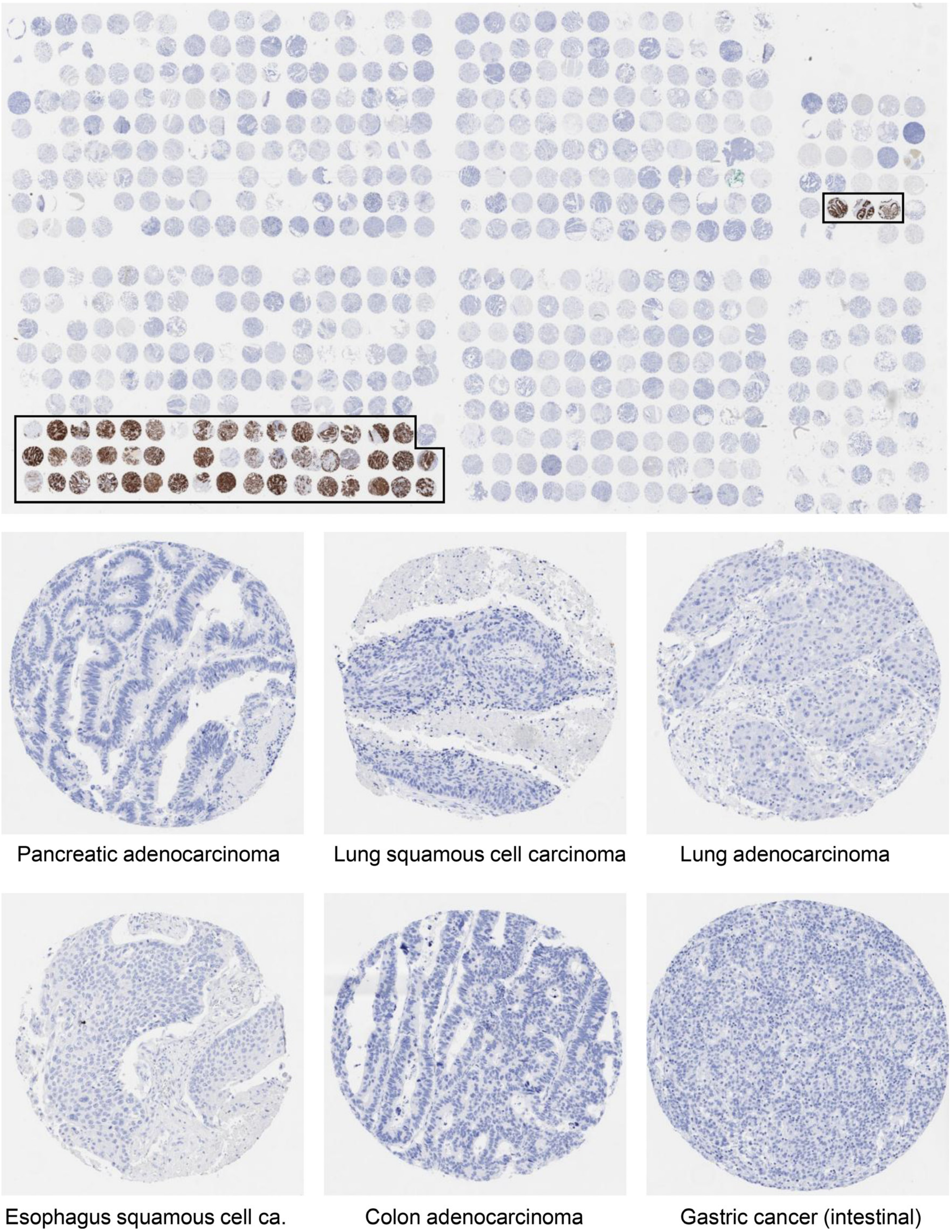

Supplementary Figure 2: Examples of PSA-negative non-prostate cancers. The position of PSA-positive prostate tissues is highlighted in the TMA section overview. All other tissue spots were taken from non-prostatic cancers.

Supplementary Table 1: Association between PSA staining (staining intensity and presence of apical predominance at 1:800 antibody dilution) and prostate cancer phenotype in all cancers and the subsets of TMPRSS2: ERG fusion negative and -positive cancers. See Supplementary Table 1

Supplementary Table 2: Association between PSA staining (staining intensity and presence of apical predominance at 1:100 antibody dilution) and prostate cancer phenotype in all cancers and the subsets of TMPRSS2: ERG fusion negative and -positive cancers. See Supplementary Table 2
